# The Ethno-Sexual Hyphen: Communicating Gay-Mizrahi Subjectivity in Israeli Workplaces

**DOI:** 10.3390/bs16081449

**Published:** 2026-08-21

**Authors:** Moshe Hajaj, Elazar Ben-Lulu

**Affiliations:** Sociology and Anthropology, Ariel University, Ariel 4070000, Israel

**Keywords:** workplace communication, organizational legibility, identity calibration, hyphenated subjectivity, DEI, gay-Mizrahi

## Abstract

This study examines how gay-Mizrahi men in Israel navigate the communicative processes through which workplace subjectivities are constructed, negotiated, and legitimized. Mizrahiness refers to a Jewish ethno-cultural position associated with Middle Eastern and North African ancestry, historically shaped by unequal relations with Ashkenazi hegemony and intersecting class, geographic, cultural, and gendered hierarchies. Based on semi-structured interviews with self-identified gay-Mizrahi men across diverse occupational sectors, this study examines how sexuality, ethnicity and professionalism are read together in everyday organizational interactions. The findings reveal three strategic mechanisms: identity calibration, hyphenated subjectivity, and compensatory professionalism. Identity calibration refers to the ongoing tuning of identity visibility, timing, intensity, and communication. Hyphenated subjectivity captures the relational process through which gayness and Mizrahiness are not simply combined but continuously negotiated in relation to social expectations and organizational norms. Compensatory professionalism describes intensified investment in excellence, credentials and self-control to counter anticipated devaluation. These mechanisms show that organizational belonging is not a static outcome of diversity or inclusion but an advanced communicative achievement. Thus, this study contributes to workplace communication and Diversity, Equity and Inclusion (DEI) scholarship by illustrating how marked workers achieve professional legibility. We argue that DEI practice must move beyond visible categories of diversity towards affective and communicative dimensions through which inequality is reproduced.

## 1. Introduction

Workplaces are not only sites of employment, mobility, evaluation, and organizational membership. They are also communicative environments in which employees are continuously read, interpreted, and positioned through everyday interactions. Names, bodies, voices, accents, gestures, sexualities, ethnicities, classed geographies, and styles of self-presentation become meaningful within organizational life because they are interpreted through shared expectations of professionalism, credibility, authority, and belonging (Acker, 2006; Y. Cohen & Haberfeld, 2007; Rivera, 2012). Workplace communication therefore extends beyond formal exchanges, managerial feedback, or institutional discourse. It also includes jokes, silences, informal conversations, assumptions, compliments, questions, and subtle cues regarding what kinds of selves are considered appropriate, competent, or legitimate. Through these communicative practices, workers learn how they are perceived, which parts of themselves can be expressed, which must be softened or withheld, and how they should present themselves in order to be recognized as professional subjects. This article begins from the premise that subjectivity at work is not merely carried into organizations by employees; it is continuously produced, negotiated, and constrained through communication.

This communicative production of subjectivity becomes particularly significant for workers whose identities are socially marked by sexuality, ethnicity, race, class, gender, or place. For employees who are perceived as aligned with dominant organizational norms, professional legitimacy may appear relatively unmarked or self-evident (Acker, 2006; Lewin-Epstein & Semyonov, 1992). By contrast, workers whose bodies, names, voices, or biographies are read as different often face an additional communicative burden: they must learn how to become professionally legible. Professional legibility refers here to the process through which workers become recognizable to others as competent, credible, appropriate, and worthy of organizational belonging. This process is rarely explicit. It is learned through repeated encounters with organizational expectations: what is praised, what is questioned, what is joked about, what remains unsaid, and what must be explained. Such practices do not necessarily indicate exclusion in its direct or formal sense. Rather, they reveal the conditional nature of belonging: the extent to which recognition depends on the worker’s ability to read the room, manage visibility, and communicate legitimacy in ways that fit dominant organizational codes (Bourassa, 2021; Jakob Sadeh & Mair, 2024; Ortlieb et al., 2021). In this article, organizational legibility functions as the central analytic concept: the desired communicative condition through which marked workers become readable as legitimate professional subjects. The three mechanisms developed later in the article are understood as the communicative means through which this legibility is pursued.

The Israeli context provides a particularly generative site for examining these processes. Mizrahiness is a historically charged social category that cannot be reduced to ethnic origin alone (Shenhav & Hever, 2012; Shohat, 1999). It refers broadly to Jews whose families migrated, or were displaced, from Middle Eastern, Arab and North African countries, but it also operates as a cultural, classed, racialized, and spatial position within Israeli society. In organizational life, however, Mizrahiness is not always explicitly named as a diversity category. It may instead appear indirectly through vocabularies of class mobility, cultural fit, professionalism, “background,” regional difference, surname, accent, family history, religiosity, masculinity, or proximity to the geographic and symbolic periphery. This indirect appearance of Mizrahiness is crucial for workplace analysis, because ethnic marking often operates through seemingly neutral organizational vocabularies: professionalism, refinement, background, mobility, fit, and institutional familiarity (Sasson-Levy & Shoshana, 2013; Avissar, 2025).

This position is inextricably linked to a long-standing struggle for recognition, rooted in decades of systemic inequality and historical grievances. Since the state’s early years, Mizrahi immigrants often faced “repressive and impatient” assimilationist policies that delegitimized their cultural heritage as “inferior” while relegating them to the socio-economic periphery. This structural deprivation was further reinforced through Mizrahi enclaves, processes of proletarianization, and persistent gaps in class mobility, education, and symbolic recognition, which have fueled generations of protest from the Wadi Salib riots of 1959 to later public struggles over ethnic discrimination (Kalman, 2020; Smooha, 2008). For the purposes of this article, this history matters because Mizrahiness enters organizational life not only as ethnic origin but as a sign read through classed geography, cultural legitimacy, family history, surname, accent, and proximity to or distance from Ashkenazi-coded norms of refinement and institutional belonging (Shohat, 1999; Sasson-Levy & Shoshana, 2013; Avissar, 2025; Daniele, 2020).

LGBTQ+ life in Israel, by contrast, has undergone significant processes of public visibility, institutionalization, and partial normalization, particularly through civil society organizations, media representations, Pride events, and organizational diversity initiatives. Yet LGBTQ+ visibility does not necessarily mean that all LGBTQ+ subjects become equally recognizable or equally included. Public and organizational representations of LGBTQ+ identity often privilege particular forms of gay subjectivity: urban, secular, liberal, middle-class, Western-oriented, and culturally legible within dominant professional environments (Hartal & Sasson-Levy, 2018; Puar, 2008; Ben-Lulu & Hajaj, 2026).

Recent LGBTQ+ workplace scholarship shows that formal visibility and institutional inclusion do not necessarily make workplaces safe. LGBTQ+ employees continue to navigate disclosure dilemmas, anticipated discrimination, heteronormative assumptions, microaggressions, and the risk of being perceived as too visible, too gender-nonconforming, or insufficiently professional (Badgett et al., 2021; Lyndon et al., 2023; Maji et al., 2024; Owens & Mills, 2025). Owens and Mills (2025) conceptualize the workplace as an “unbounded” space in which discrimination and minority stress are not confined to the physical workplace but travel across time, space, home life, and mental health. Recent qualitative research on LGBTQ+ professionals further shows that disclosure is not a single event but an ongoing assessment of environmental signals, safety, professionalism, and risk (Nelson et al., 2026; Galante, 2026; Selen et al., 2026; Buizer et al., 2025). Visibility may produce pride and connection, but it can also generate additional labor, minority taxation, and disillusionment when inclusion remains performative.

Despite growing scholarly attention to workplace communication, organizational inequality, LGBTQ+ inclusion, and ethnic or racialized difference at work, these bodies of literature often remain analytically separated. LGBTQ+ workplace research has contributed important insights into disclosure, concealment, discrimination, identity management, and workplace belonging (Clair et al., 2005; Ragins et al., 2007; Maji et al., 2024; Nelson et al., 2026). By contrast, scholarship on ethnic and racialized inequality has shown how organizational norms reproduce exclusion through assumptions about professionalism, cultural fit, credibility, and mobility (Khattab & Miaari, 2013; Swirski & Konor, 2002; Acker, 2006; Rivera, 2012). In the Israeli context, studies of Mizrahiness have examined its historical, cultural, classed, and spatial dimensions, yet its communicative presence within organizational life remains insufficiently explored (Shohat, 1999; Sasson-Levy & Shoshana, 2013; Avissar, 2025; Daniele, 2020). What remains less developed is an account of how sexuality and Mizrahiness are read together in ordinary workplace communication and how workers navigate the combined communicative demands produced by this relational reading (Crenshaw, 2013; Bowleg, 2012; Hartal & Sasson-Levy, 2018).

Based on semi-structured interviews with self-identified gay-Mizrahi (in this study ‘gay’ refers to self-identified homosexual men) men in Israel, this work asks: How do gay-Mizrahi men describe and interpret the communicative processes through which their workplace subjectivities are shaped, negotiated, and rendered professionally legitimate? In answering this question, the article contributes to workplace communication studies by showing that communication is not merely a channel through which identity is expressed but a mechanism through which minority subjectivities become readable, credible, or problematic. The article’s central contribution is to show that organizational legibility is produced through subterranean communicative labor. It further contributes to organizational inequality scholarship and to Mizrahi and LGBTQ+ studies by conceptualizing gay-Mizrahi identity not as an additive combination of two categories but as a hyphenated subjectivity produced through the relational reading of sexuality, ethnicity, masculinity, classed geography, and professionalism in Israeli workplaces (Bowleg, 2012; Crenshaw, 2013; Hartal & Sasson-Levy, 2018).

The article’s conceptual framework is organized hierarchically. Organizational legibility is the central analytic concept and refers to the communicative condition in which workers become recognizable as legitimate professional subjects. Subterranean labor refers to the hidden communicative work required to pursue this legibility under non-neutral organizational conditions. Identity calibration, hyphenated subjectivity, and compensatory professionalism are the three mechanisms through which this labor is enacted in everyday workplace communication. First, we develop this conceptual framework. Second, we detail the qualitative methodology involving interviews with ten gay-Mizrahi men and reflect on the first author’s role as an intimate insider. Third, we present the findings through these three mechanisms. Finally, we discuss the study’s implications for workplace communication and DEI practice.

### Workplace Communication, Identity, and Organizational Legibility

The theoretical framework begins from the claim that organizational subjectivity is communicatively produced. Rather than treating identity as an individual possession that workers simply disclose, conceal, or manage, this article conceptualizes organizational legibility as a relational achievement produced between workers and the organizational codes through which they are read (Clair et al., 2005; Ragins et al., 2007; Acker, 2006; Rivera, 2012).

To show how workplace subjectivity is communicatively constructed, and how marked workers participate in producing themselves as professionally legible subjects, we distinguish organizational legibility from three adjacent concepts: visibility, recognition, and cultural fit. Visibility often refers to the binary of disclosure versus concealment, particularly in relation to stigmatized or minoritized identities (Button, 2004; Clair et al., 2005; Ragins et al., 2007). Recognition typically concerns formal institutional acknowledgments, such as DEI policies, inclusion statements, or surface-level diversity initiatives (Ahmed, 2012; Nittrouer et al., 2025; Roberson, 2006; Shore et al., 2011). Cultural fit, by contrast, is often treated as a pre-existing alignment between the worker and organizational norms (Acker, 2006; Rivera, 2012). Legibility differs from all three because it captures the mundane interactional labor required to be perceived as a competent and credible professional subject. We define legibility as a relational and political process: the communicative register through which a worker’s body, voice, and biography are rendered meaningful and appropriate within dominant organizational codes. This production is inherently uneven, as workers marked by sexuality, ethnicity, or class bear a particular communicative burden to align themselves with professional norms that are never neutral but embedded in histories of power (Acker, 2006; Izak et al., 2024; Oluyadi & Dai, 2023; Shore et al., 2011; Stevens & Connelly, 2024). This distinction is important because it shifts the analytic focus from whether organizations formally recognize difference to how workers must make themselves intelligible within dominant professional codes.

Two strands of research are particularly important for developing this argument: scholarship on ethnicized organizational norms and scholarship on LGBTQ+ workplace vulnerability. Research on ethnic and racialized inequality in organizations shows that workplaces often reproduce social hierarchies through seemingly neutral norms of professionalism, cultural fit, evaluation, and advancement. Ethnicized and racialized workers may be formally included while still encountering microaggressions, assumptions about competence, limited access to informal networks, and pressure to adapt speech, demeanor, or self-presentation to dominant expectations (Acker, 2006; Rivera, 2012). In the Israeli context, scholarship on Mizrahiness and Ashkenazification ((hishtaknezut) refers to the adoption by Mizrahim of practices associated with the dominant European-Ashkenazi habitus in an attempt to pass as normatively non-ethnic and improve their prospects for social and economic mobility (Sasson-Levy & Shoshana, 2013, pp. 448–449)) shows how ethnic hierarchy may be reproduced through cultural codes of refinement, whiteness, classed geography, and the ability to pass as “non-ethnic” or culturally neutral (Sasson-Levy & Shoshana, 2013). More recent work further emphasizes that Ashkenaziness often operates not as a marked ethnic identity but as a transparent norm associated with symbolic capital, institutional familiarity, and cultural neutrality (Avissar, 2025). Critical scholarship on Mizrahi positionality likewise shows that Mizrahiness occupies an ambivalent position of inclusion and marginalization within Israeli society, where practices of Ashkenazification and “acting white” may function as routes toward legitimacy under Ashkenazi-coded standards of power and respectability (Daniele, 2020). Thus, ethnic belonging at work is not secured by employment alone; it is negotiated through everyday organizational readings of names, accents, biographies, bodies, and professional styles.

Recent LGBTQ+ workplace scholarship similarly shows that formal visibility and institutional inclusion do not necessarily make workplaces safe. LGBTQ+ employees continue to navigate disclosure dilemmas, anticipated discrimination, heteronormative assumptions, microaggressions, and the risk of being perceived as too visible, too gender-nonconforming, or insufficiently professional (Badgett et al., 2021; Maji et al., 2024; Owens & Mills, 2025). This literature is developed further in the theoretical framework, where recent studies on disclosure, workplace safety, and queer visibility are discussed in greater detail.

This literature is important not only because it documents discrimination but because it shows that workplace safety is communicatively assessed. LGBTQ+ workers read environmental signals, institutional language, colleague reactions, managerial cues, and broader sociopolitical climates in order to decide whether, when, and how identity can appear at work (Clair et al., 2005; Ragins et al., 2007; Nelson et al., 2026). Even organizations that present themselves as gay-friendly may regulate queer visibility through expectations regarding voice, body, gender performance, and emotional tone (Galante, 2026). These studies suggest that workplace safety is not secured by formal inclusion alone. Rather, it is continuously negotiated through everyday communication, interpretation, and self-presentation (Owens & Mills, 2025).

Bringing these studies together reveals the need to move beyond single-axis accounts of workplace identity. LGBTQ+ workplace scholarship has shown how sexuality is managed through disclosure, concealment, visibility, and safety assessment (Clair et al., 2005; Maji et al., 2024; Ragins et al., 2007), while research on ethnic and racialized inequality has shown how professionalism and legitimacy are shaped by cultural fit, classed mobility, and racialized or ethnicized organizational norms (Acker, 2006; Rivera, 2012). However, for gay-Mizrahi men, sexuality and Mizrahiness are not two separate attributes that can simply be added together. They are relationally interpreted in specific interactions: gayness may soften Mizrahiness, Mizrahiness may ground gayness, and both may intensify the demand to appear competent, controlled, and professionally legitimate (Bowleg, 2012; Crenshaw, 2013; Hartal & Sasson-Levy, 2018; Ben-Lulu & Hajaj, 2026). The present article therefore treats gay-Mizrahi subjectivity as hyphenated: a communicative formation produced through the mutual reading of sexuality, ethnicity, masculinity, classed geography, and professionalism.

We use the term identity calibration to describe the continuous, situational tuning of identity visibility, intensity, timing, and communicative form. Calibration extends existing discussions of disclosure, concealment, passing, and covering by emphasizing not only whether identity is revealed or hidden but how its visibility is adjusted in relation to organizational cues, interlocutors, hierarchy, and perceived risk (Clair et al., 2005; Maji et al., 2024; Ragins et al., 2007). In this sense, identity calibration is not a one-time decision but an ongoing communicative practice through which workers attempt to become professionally legible without exposing themselves unnecessarily to misrecognition, stereotyping, or intrusive explanation.

Hyphenated subjectivity moves beyond additive understandings of intersectionality by focusing on the communicative relations between identities. The emphasis here is not only on the coexistence of multiple identities but on the work through which one identity changes the meaning, risk, or usefulness of another in specific workplace encounters (Bowleg, 2012; Crenshaw, 2013). For gay-Mizrahi men, gayness and Mizrahiness may mutually soften, translate, authorize, or intensify one another. This is particularly important in the Israeli context, where both queer visibility and Mizrahi belonging are shaped by dominant norms of Ashkenazi respectability, secular liberal gay subjectivity, classed geography, and cultural legibility (Daniele, 2020; Hartal & Sasson-Levy, 2018; Sasson-Levy & Shoshana, 2013). Hyphenated subjectivity therefore captures the relational work through which participants learn when identity can become a resource, when it becomes a risk, and how the relation between identities may produce professional connection, intimacy, or distance.

Compensatory professionalism refers to intensified investment in excellence, credentials, self-control, and professional polish as a way of countering anticipated devaluation. This mechanism builds on research showing that marked workers may be required to provide stronger evidence of competence in order to be read as credible, legitimate, or worthy of advancement (Acker, 2006; Rivera, 2012; Stevens & Connelly, 2024). In the Israeli context, this dynamic also resonates with scholarship on Mizrahi mobility, gendered and ethnicized professionalism, and the demand to convert achievement into proof of worth (Karazi-Presler, 2021; Sasson-Levy & Shoshana, 2014; Tsalach, 2013). For gay-Mizrahi men, compensatory professionalism captures how achievement becomes entangled with defense: professional excellence functions not only as aspiration but also as a communicative shield against ethnic, sexual, classed, or peripheral stigma. This mechanism also links professional performance to minority stress, because the need to become exceptionally prepared, controlled, or credentialed may respond to anticipated rejection or devaluation while carrying emotional costs through vigilance, exhaustion, and the continuous production of proof (Holman, 2018; Nelson et al., 2026; Owens & Mills, 2025). In this sense, compensatory professionalism may function as a pathway through which subterranean labor becomes emotionally and psychologically costly.

These concepts form a hierarchical framework. Organizational legibility is the communicative goal: becoming recognizable as a legitimate professional subject. Subterranean labor is the hidden work required to pursue this goal under non-neutral organizational conditions. Identity calibration, hyphenated subjectivity, and compensatory professionalism are the mechanisms through which this labor is enacted in everyday workplace communication. The following section explains how the interview data were collected and analyzed in order to develop this framework empirically.

## 2. Methodology

This study employs a qualitative interpretive research design. This approach is suitable because the study seeks to understand how participants narrate and make sense of their everyday communicative experiences rather than measuring the prevalence of discrimination. The study is framed within the Communicative Constitution of Organizations (CCO) perspective, which views organizations as emergent through ongoing interactions (Izak et al., 2024).

Participants were ten self-identified gay-Mizrahi men in Israel, aged 25 and above. All participants had at least three years of professional experience. This criterion was established to ensure participants had moved beyond initial organizational socialization and could reflect on repeated workplace encounters, professional expectations and identity-related dynamics over time (Ibarra, 1999; Ng & Feldman, 2010). Participants were recruited through snowball sampling and social media calls, with an effort to include diverse occupational sectors. These settings differ in their organizational hierarchies, professional norms, communicative expectations, and gendered cultures. For example, academia and high-tech often place strong emphasis on linguistic polish, credentials, and symbolic professionalism, while construction, food and beverage, and factory management may involve more direct hierarchies, embodied forms of authority, and differently coded expectations of masculinity and managerial presence. The study does not offer a systematic cross-sector comparison. Rather, occupational diversity was used to examine how gay-Mizrahi men narrated the pursuit of professional legibility across varied organizational contexts. Accordingly, the findings are presented as recurring patterns within this sample, not as evidence that the same mechanisms operate identically across sectors.

Data were collected through semi-structured depth interviews. Due to geographical constraints and participant preference, interviews were conducted either face-to-face or via synchronous video conferencing (Zoom), a method recognized as effective for building rapport in qualitative research (Archibald et al., 2019; Weller, 2017). Interviews lasted between 60 and 90 min and were audio-recorded and fully transcribed. The interview guide focused on workplace histories, everyday interactions, experiences of belonging and exclusion, identity visibility, professional legitimacy, and the ways participants interpreted the relation between gayness and Mizrahiness at work. Participants were told that the study sought to understand not only experiences of discrimination or exclusion but also how they experience, interpret, and navigate everyday organizational life as gay-Mizrahi men.

The study was approved by the Institutional Ethics Committee for Non-Clinical Research Involving Human Participants at Ariel University (approval no. AU-SOC-EBL-20260323). Informed consent was obtained from all participants. Participants were informed of the study’s aims, the voluntary nature of participation, their right to withdraw, and the measures taken to protect confidentiality. All names were replaced with pseudonyms, and identifying details were removed or modified where necessary to protect participant anonymity.

The analysis was conducted using reflexive thematic analysis (Braun & Clarke, 2022), supported by a systematic approach to conceptual model development in qualitative research (Naeem et al., 2023). The process involved repeated listening to the interviews, close reading of transcripts, and analytic memo-writing after each interview. Initial coding focused on workplace interactions, identity visibility, professional legitimacy, ethnic marking, disclosure, humor, silence, and self-presentation. As the interviews progressed, and particularly around the seventh interview, recurring patterns began to emerge around the management of visibility, the relational use of gayness and Mizrahiness, and the intensified demand to prove professional worth. Subsequent interviews were used to probe, refine, and complicate these emerging patterns. The initial codes were gradually clustered into three interpretive themes: identity calibration, hyphenated subjectivity, and compensatory professionalism. These themes were then integrated into the conceptual model developed in this article, in which organizational legibility is understood as the communicative goal and subterranean labor as the hidden work required to pursue it.

The final sample was considered adequate for the conceptual aim of the study because the participants were highly specific to the research question, all met the inclusion criteria, and the interviews generated rich and detailed accounts of workplace communication, identity negotiation, and professional legitimacy. The study does not claim statistical representativeness, population-level generalization, or sectoral saturation. Rather, the sample provided sufficient conceptual depth to develop an interpretive framework grounded in recurring patterns across participants’ narratives. The final interviews were therefore used not simply to add cases but to test, refine, and complicate the emerging themes.

Interviews were conducted in Hebrew and translated into English by the author. Translation was treated as a hermeneutic and interpretive procedure rather than a merely technical transfer between languages. During translation, the author checked quotations against the Hebrew transcripts in order to preserve tone, meaning, and culturally specific references. Terms such as Mizrahiness, Ashkenazification, periphery, and “white language” were retained, transliterated, or translated with explanatory wording where necessary, in order to avoid flattening their sociological meanings.

The authors’ positionalities shaped the research process, access to the field, and interpretation of the interview material. Both authors are gay-Mizrahi scholar. The first author conducted the fieldwork and brought to the interviews both academic training and extensive practical experience in LGBTQ+ community work and organizational diversity initiatives. This position facilitated trust, rapport, and a shared cultural vocabulary with participants but also required reflexive caution. The first author avoided treating shared references as self-evident and asked participants to clarify the meanings they attached to ethnic labels, geographic references, family histories, and workplace experiences. The second author is an anthropologist whose research focuses on LGBTQ+ Jews in Israeli and American contexts, with particular attention to identity, community, ethnicity, representation, reflexivity, and autoethnography. His involvement added a complementary interpretive perspective and supported reflexive dialogue regarding the relationship between emic and etic perspectives, insider knowledge, cultural interpretation, and analytical distance. Positionality was therefore treated not as data in itself but as an interpretive condition that shaped access, listening, analysis, and the ongoing reflexive monitoring of the study (Ben-Lulu, 2022, 2024a, 2024b, 2025; Marcus, 2005; Taylor, 2011).

## 3. Findings

The findings are organized around three interrelated mechanisms: identity calibration, hyphenated subjectivity, and compensatory professionalism. Together, they show how gay-Mizrahi men’s workplace subjectivities are constructed as professionally legible through everyday workplace communication. Participants described organizational life as a space in which identity is continuously read, adjusted, translated, and evaluated (Acker, 2006; Izak et al., 2024). Their narratives show how sexuality, Mizrahiness, masculinity, classed geography, and professionalism become meaningful through situated interactions with managers, colleagues, workers, clients, and institutional expectations.

These mechanisms are not separate stages or fixed categories. Instead, they capture different dimensions of the same broader process: becoming professionally legible as a gay-Mizrahi subject within Israeli workplaces. Identity calibration refers to participants’ ongoing adjustment of identity visibility, tone, language, and self-presentation in response to organizational cues (Maji et al., 2024). Hyphenated subjectivity captures the ways gayness and Mizrahiness are not merely added together but mutually shape one another in workplace interactions (Crenshaw, 2013; Hartal & Sasson-Levy, 2018). Compensatory professionalism describes the intensified investment in excellence, credentials, self-control, and professional performance through which participants sought to communicate legitimacy and counter anticipated devaluation (Karazi-Presler, 2021; Stevens & Connelly, 2024). In what follows, we examine each theme through participants’ narratives, showing how workplace communication produces both possibilities for agency and conditions of constraint.

### 3.1. Identity Calibration: Learning to Fit What Is Possible

Identity calibration refers to the ongoing work through which participants read organizational settings and adjust the visibility, intensity, timing, and communicative form of their identities. Participants did not present gayness or Mizrahiness as stable attributes that were simply expressed or hidden. Instead, they described identity as something that had to be continuously calibrated in relation to who was present, what kind of organization they were in, what was at stake, and how they believed their identities would be interpreted. This calibration included decisions about when to speak, when to remain silent, when to use identity as a resource, and when to soften, delay, or translate aspects of the self. Across the interviews, calibration appeared not only in explicit decisions about disclosure but also in speech, bodily presentation, professional tone, digital self-presentation, and the management of names, accents, and ethnic signs.

Yahli, 39, an industrial engineering and information systems professional in the high-tech industry, articulates this process as an ongoing “game” with identity:

“You are constantly in a game with the identities you have in order to try to fit what is possible. […] I have completely internalized certain codes of behavior in different places, and I use them in everyday life in order to move through them in the best way. When you understand who is standing in front of you, there are all kinds of doors you can enter through, and you choose the identity through which the passage will be the smoothest.”

Yahli plays a conscious game with his identities. He learns the codes and act upon them. He seems to be constantly busy with what aspects of his identity needs the front. The usage of the metaphor “doors” help us understand that it is not a one-sided route but a two-way path as if saying two need to play this game. The worker must identify which identity will allow for the “smoothest” passage. This does not suggest deception or inauthenticity. Rather, it shows how gay-Mizrahi workers learn to navigate environments in which not all parts of the self are equally available, equally safe, or equally professionally useful (Maji et al., 2024). Yahli’s account therefore highlights the double character of calibration: it is an expression of agency, but it is also a response to unequal communicative conditions. Those whose identities are already aligned with dominant organizational codes are less often required to ask which part of themselves can enter the room first.

Chaim, 39, a manager in the food and beverage industry, describes a similar process but frames it more explicitly as the strategic use of identity:

“Use your identities wisely; know when it works in your favor. […] Know when to pull them out, when it helps you, and do not just put them there without using them. At higher levels […] they see a white face before I even open my mouth, and I already think that it gives them some comfort, or that they are happier.”

Chaim’s narrative sharpens the instrumental dimension of identity calibration. He knows he passes as a white-Ashkenazi man, and he uses this to his advantage. Chaim perceives that his proximity to whiteness appears to ease his access to senior organizational spaces. His formulation “use your identities wisely” suggests that he puts his physical attributes to work transforming identities into communicative resources whose value changes across organizational situations (Maji et al., 2024; Ragins et al., 2007). Yet the quotation also exposes the racialized and ethnicized conditions under which such resources operate. Chaim does not assume that identity will be interpreted neutrally. In his account, the body, face, perceived ethnicity, and assumed proximity to whiteness already communicate something to the organization. This can be read as a form of aesthetic labor where physical appearance becomes an organizational resource (Oluyadi & Dai, 2023; Stevens & Connelly, 2024). In this sense, identity calibration is not only about what participants say but about how they anticipate being read. The need to “pull out” identities only when useful reveals a workplace order in which belonging is conditional upon the correct timing, intensity, and framing of difference (Cui & Song, 2024; Shore et al., 2011).

Shmuel, 41, who works in academia and education and holds a doctoral degree, extends the logic of calibration from the visible body to the nuance linguistic performance.

He recognizes that achieving organizational legibility is not merely a matter of appearance but a meticulous management of speech patterns, vocabulary, and the display of specific cultural knowledge. For Shmuel, calibration involves mastering the unwritten codes of language to ensure he is “counted” as a legitimate subject within the professional hierarchy. He explains:

“I internalized the correct way of speaking, the way of thinking that one is supposed to think in academia. I learned to speak the academic language, the ‘white’ language, so that my ideas would sound legitimate and they wouldn’t categorize me in advance. This is identity management where you understand that if you don’t speak on their ‘frequency,’ they won’t listen to the content of what you have to say.”

Shmuel’s account shifts identity calibration from the management of visible identity to the management of linguistic legitimacy. His emphasis on “the correct way of speaking” and “the academic language” shows that organizational and academic spaces do not merely evaluate ideas; they evaluate the communicative form through which ideas are presented (Ramjattan, 2019; Izak et al., 2024). The phrase “white language” is analytically significant because it links legitimacy to dominant cultural codes. Shmuel does not claim that his ideas lack value. Rather, he suggests that without speaking on the correct “frequency,” the content itself may not be heard. This makes calibration a condition of audibility (Sasson-Levy & Shoshana, 2013; Shem-Tov, 2025). In order to become intellectually and professionally legible, he must translate himself into a language that will not trigger prior categorization (Flores & Rosa, 2015; Tsalach, 2013). It is not only the body or biography that must be calibrated but also the very mode through which thought becomes recognizable as legitimate.

Oren, 38, a project manager in the construction industry,

Oren clarifies that his calibration practices are not motivated by internal shame or a rejection of his cultural roots. Instead, he views the management of his digital persona as a strategic functional necessity intended to pre-emptively bypass ethnic categorization and prevent unsolicited social inquiries that he deems irrelevant to his professional role. He says:

“I don’t tend to say my last name in the daily routine at the office; if you notice, on Zoom it just says ‘Oren L.’ This stems from the desire to avoid an ethnic discourse where they categorize me or start asking questions about my origins. There is a desire in this to maintain a sort of ‘white’ persona, of something neutral that doesn’t require explanations.”

Oren’s account illustrates how the mechanism of identity calibration operates through seemingly mundane digital infrastructures. By shortening his surname on Zoom to “Oren L.,” he does not merely hide information but actively manages the conditions of his legibility to avoid unsolicited ethnic inquiries (Maji et al., 2024; Ramjattan, 2019; Tsalach, 2013). This underscores a critical asymmetry in organizational life: the neutral persona he seeks is inextricably linked to the absence of ethnic marking (Ahmed, 2007; Sasson-Levy & Shoshana, 2013). Ultimately, we see that digital communication does not erase identity work; instead, it produces new sites for calibration labor, where a name on a screen becomes a tool to control the timing and conditions of entering ethnic discourse (Izak et al., 2024; Oluyadi & Dai, 2023; Stevens & Connelly, 2024).

These narratives show that identity calibration operates across multiple communicative registers: strategic identity use, anticipatory bodily reading, linguistic adaptation, and digital self-presentation. Yahli and Chaim describe the need to read situations and decide which identity can move most smoothly through a given organizational door. Shmuel shows how legitimacy depends on speaking in a recognized professional or academic frequency. Oren demonstrates how even a surname on Zoom can become an ethnic marker requiring management. Across these accounts, calibration enables participants to act strategically and move within organizations, but it also reveals the unequal labor required to become professionally legible. The worker is not simply asked to perform a role; he is asked to manage the communicative conditions under which his identity will be heard, seen, and judged.

### 3.2. Hyphenated Subjectivity: Gayness, Mizrahiness and Relational Legibility

Hyphenated subjectivity captures the ways participants described gayness and Mizrahiness not as separate identity categories but as mutually shaping positions (Crenshaw, 2013; Hankivsky, 2014). The hyphen in gay-Mizrahi identity does not merely connect two social categories; it marks a relational space in which one identity may soften, intensify, complicate, authorize, or translate the other (Hartal & Sasson-Levy, 2018; Shohat, 2002). Participants often narrated situations in which Mizrahiness affected how their gayness was read, or in which gayness altered the meaning of masculinity, intimacy, authority, and professional belonging (Maji et al., 2024; Stevens & Connelly, 2024). This theme therefore moves beyond an additive understanding of intersectionality. It asks how the two sides of the hyphen communicate with, through, and against one another in everyday workplace life.

Chaim, 39, a manager in the food and beverage industry, describes Mizrahiness as something that changes the way his gayness is perceived:

“It helps me because I do not pass only as an elitist Ashkenazi gay man; Mizrahiness helps me soften that homosexuality and that elitism. It gives me a feeling that I am more accessible to people, as if I am here, I am grounded, I am from the neighborhoods. Mizrahiness allows me for a moment to take off that mask and say: we are not millionaires, we are from the periphery, we are this and that.”

Chaim’s narrative demonstrates that Mizrahiness is not solely a social disadvantage but can function as a strategic communicative resource, fostering a sense of accessibility and social familiarity. In many organizational settings, gay identity is frequently read through assumptions of Ashkenazi elitism or a cosmopolitan distance from “ordinary” workers (Abutbul-Selinger, 2022; Hartal & Sasson-Levy, 2018; Puar, 2008). Mizrahiness intervenes in this perception, effectively softening the subject by attaching his sexuality to the warmth and social proximity of the neighborhood. Yet, as we observe, this resourcefulness remains conditional (Cui & Song, 2024). Mizrahiness becomes an organizational asset only when it manifests in a form that is readable as authentic and non-threatening, a practice that aligns with the performance of transracial aesthetic labor (Ramjattan, 2021). It is accepted specifically when it facilitates relational ease. This account illustrates the ambivalence of hyphenated subjectivity: one identity can authorize or translate the other but only within the boundaries of what the organization can recognize as useful or comfortable.

Avner, 36, a manager in a fashion factory, illustrates a different form of hyphenated subjectivity, in which Mizrahiness operates as cultural knowledge that enables workplace connection:

“I understand the culture they live in. For example, one of the workers, his daughter got married, so the next day he brought a plate of sweet cookies. I know this; it is not foreign to me. […] It helps me create a connection with them that the other managers, the kibbutzniks, do not always manage to create. All along the way, I look at them at eye level.”

Avner utilizes his cultural knowledge into his situated role. Not only Avner demonstrates the way in which cultural familiarities form connections—he also demonstrates the gap between him and his peers by referring to them as “Kibbutzniks”. His retrospective on his connections with the employees leads him to tear up mid-interview. He is able to read gestures, family practices, food, celebration, and informal codes that other managers may overlook or misinterpret (Izak et al., 2024; Misgav, 2014). The plate of cookies becomes more than a cultural detail; it is a communicative event through which workplace relationships are formed. Avner’s ability to recognize its meaning allows him to produce closeness, respect, and managerial legitimacy (Farndale et al., 2015; Shem-Tov, 2025). At the same time, the comparison to “the kibbutzniks” positions him against a different organizational habitus associated with Ashkenazi, rural-socialist, or dominant Israeli institutional culture (Gigi & Tzfadia, 2024). His claim to look at workers “at eye level” suggests that hyphenated subjectivity may enable a different mode of authority: not distance but relational recognition. Here, Mizrahiness does not undermine professionalism; it becomes part of how professionalism is practiced.

Yoav, 38, who works in education, describes how gay identity can authorize forms of intimacy with heterosexual male colleagues that might otherwise be read as socially or sexually threatening:

“There is still that clear boundary of ‘I am gay and he is straight,’ and then we can lean on each other in the teachers’ room and it will look fine. It won’t look sexual, it will look humorous because the boundaries have already been predefined through my identity. This allows me to create safe intimacy with straight colleagues that in another way could have been perceived as threatening.”

For Yoav, embodied interactions function as primary communicative channels that foster a sense of security and safety between him and his colleagues. In heteronormative masculine environments, physical closeness between men is often regulated by social anxieties regarding sexuality and masculinity (Embrick et al., 2007; Hartal & Sasson-Levy, 2018; Maji et al., 2024). However, Yoav’s gay identity acts as a mechanism that predefines the interactional boundaries, effectively translating bodily contact into a safe and humorous form of collegial intimacy. This suggests that identity is not a static description of the self but an active tool that organizes how others are permitted to read and interpret a specific encounter. In Yoav’s account, gay identity helps organize the meaning of physical closeness, allowing it to be read as humorous and safe rather than threatening. What might otherwise appear as a potential vulnerability becomes, in this specific interactional context, a resource for building social connection with heterosexual colleagues. (Izak et al., 2024; Schönauer et al., 2025; Stevens & Connelly, 2024).

Yahli, 39, who works in the high-tech industry, similarly articulates gay identity as a relational and professional resource. Yahli recognizes that his gay identity functions as a versatile communicative asset that allows him to navigate gendered hierarchies by presenting a version of masculinity that is both accessible and non-confrontational. He strategically utilizes his “gayness” because it is perceived as “soft” enough to foster deep relational trust with women while remaining “non-threatening” to the heterosexual men in his professional circle, thereby securing his position across various social groups:

“As gay men in the workforce, we have an edge. I have an excellent relationship with the men, and at the same time, women feel very comfortable talking to me on a personal level because of my identity. I use my homosexuality with women to create this bonding, in order to later pave a stronger professional path.”

Yahli explicitly frames gayness as an “edge” in workplace relations (Abutbul-Selinger, 2022; Maji et al., 2024). His account complicates readings of minority identity solely through disadvantage, exclusion, or stigma. Instead, he expresses an active communicative agency through which gay identity becomes a tool for producing trust, intimacy, and professional opportunity (Schönauer et al., 2025; Shore et al., 2011). The relationship with women is especially significant: Yahli suggests that his gayness allows him to be perceived as emotionally available, non-threatening, and suitable for personal conversation. This personal bonding intertwined with professional advancement; it becomes part of how a “stronger professional path” is built (Farndale et al., 2015; Maji et al., 2024). At the same time, this advantage is not neutral. It relies on recognizable cultural assumptions about gay men as safe, emotionally attuned, or socially accessible to women. Yahli’s narrative therefore shows both the resourcefulness and the constraint of identity-based legibility. His identity can open doors, but it does so through pre-existing scripts about gayness, gender, and relational labor.

These narratives demonstrate that hyphenated subjectivity functions as a relational mechanism through which identity categories mutually authorize and translate one another. Rather than a simple additive identity, the hyphen operates as a site of communicative labor where participants negotiate professional legibility. We see this in the way Mizrahiness is leveraged by Chaim and Avner to soften perceived elitism or establish managerial legitimacy through cultural fluency. Conversely, Yoav and Yahli utilize gay identity to redefine the boundaries of masculine intimacy or build professional trust. While these practices afford a degree of agency, they also reveal a regulatory constraint: organizational belonging remains conditional, achieved primarily by aligning the self with recognizable scripts—such as the ‘grounded’ Mizrahi or the ‘safe’ gay colleague.

### 3.3. Compensatory Professionalism: Excelence as Communicative Shield

We define compensatory professionalism as an intensified investment in excellence, credentials, self-control, and professional polish used to pre-emptively neutralize anticipated devaluation. Participants repeatedly suggested that being professionally “good enough” was not always sufficient. For some, professional life required becoming more prepared, more educated, more careful, more polished, or more emotionally controlled in order to counter the possibility that they would be read through ethnic, sexual, or classed stigma. This mechanism does not frame ambition or achievement as artificial. Rather, it shows how achievement becomes entangled with defense: professionalism is not only a career practice but also a communicative shield through which marked subjects attempt to pre-empt suspicion, disappointment, or stereotypical reading (Karazi-Presler, 2021; Stevens & Connelly, 2024). The following narratives show how compensatory professionalism appears in different biographical and organizational contexts: as a response to emotional wounds, as academic armor, as intergenerational Mizrahi pride, and as reliance on meritocratic metrics.

Yoav, 38, who works in education, conveys this compensatory structure through growing pains. As an education professional, it seems Yoav is mending injustices he endured as a child by holding this position. Those childhood encounters still lives in him and help shape the professional he is:

“This feeling that I need to be the best in order to compensate for my identity always accompanies me, since childhood. I act this way so that they will not be disappointed in me and so that they will be proud of me and love me despite who I am. […] These things stay with you; these wounds shape who you are inside the world of work.”

Compensatory professionalism is not merely a workplace strategy developed in response to a specific organization (Karazi-Presler, 2021). It is connected to a longer social history of encountering situations in which identity may produce disappointment, distance, or conditional affection. Using ‘despite’ is crucial. Yoav does not say that he wants to be loved for who he is but ‘despite’ who he is. Professional excellence therefore becomes a way to neutralize anticipated loss (Cui & Song, 2024; Karazi-Presler, 2021). In the workplace, this takes the form of intensified effort: being the best not only as aspiration but as protection (Maji et al., 2024). His narrative links subjectivity, affect, and professionalism. The ‘wound’ is not left outside the organization; it enters work as a demand for achievement, control, and legitimacy. Compensatory professionalism thus shows how the emotional history of markedness can be translated into professional performance.

Shmuel, 41, who works in academia and education and holds a doctoral degree, provides another version of this process, in which academic credentials become a form of armor:

“The fact that I completed a doctorate was a very conscious move. […] I will achieve what others cannot achieve. I need these degrees so that they will not look at me only through my Mizrahiness; without them, I would have been stuck in the places where my uncles were stuck. […] The degree is what allows me to cross the glass ceiling.”

Shmuel’s narrative frames education not only as mobility but as protection against being reduced to Mizrahiness. The doctorate functions as symbolic capital but also as a shield. It allows him to be read differently, to cross boundaries that he imagines were closed to earlier generations in his family (Sasson-Levy & Leon, 2022; Shem-Tov, 2025). The reference to his uncles situates professional achievement within an intergenerational Mizrahi history of blocked mobility (U. Cohen & Leon, 2024; Shem-Tov, 2025). The degree becomes a communicative object: it tells the organization how to read him. It says competence, legitimacy, distinction, and exceptionality. Yet this form of mobility also reveals the persistence of inequality (Yaish, 2001). If credentials are needed in order “not to be looked at only” through Mizrahiness, then professional recognition remains conditional upon additional proof. Shmuel’s success does not cancel the stereotype; it works against the possibility of being captured by it.

Daniel, 45, who works in communications, recounts excellence as both personal confidence and an intergenerational Mizrahi project of reversal:

“I always grew up with the consciousness that I am brilliant; I skipped a grade and was the kid everyone copied from. This was rooted in my father’s Mizrahi pride; he wanted to show that his son surpasses all the Ashkenazim. I felt that no one could tell me I am ‘less than’ because I came from a moshav in the periphery, because my excellence was bulletproof.”

Daniel’s account adds an explicitly intergenerational dimension to compensatory professionalism. Excellence is not described only as an individual trait or personal achievement but as a family project shaped by Mizrahi pride, ethnic hierarchy, and the desire to reverse a social order in which Ashkenazim are imagined as the normative standard of success (U. Cohen & Leon, 2024; Shem-Tov, 2025). His father’s wish to show that his son “surpasses all the Ashkenazim” situates achievement within a collective history of comparison, injury, and aspiration. Academic and professional success become a shield that makes it impossible, or at least more difficult, for others to read him as “less than” because of peripheral social positioning or Mizrahi background (Sasson-Levy & Leon, 2022). This quotation therefore shows how compensatory professionalism may be inherited, cultivated, and emotionally charged long before the worker enters a specific workplace (Yaish & Gabay-Egozi, 2019). By the time Daniel arrives in organizational life, excellence already functions as a communicative defense: it announces that he cannot be reduced to the social meanings attached to periphery, ethnicity, or classed location.

Beni, 39, who works in the high-tech industry, connects professional achievement to earlier experiences of social vulnerability and to the promise of meritocratic recognition:

“I was a child who suffered from social boycotts, bullying, and humiliation… and I absolutely needed the Atuda [academic reserve] to survive the army afterwards. It tempered me and prepared me for life itself. In the university, in the end, the grade speaks—it doesn’t matter if your last name is Buzaglo or Cohen; in the end, the grade is what counts.”

Beni’s narrative reveals how compensatory professionalism can emerge from the relationship between social injury and the search for a system that appears to offer objective recognition. His experiences of boycotts, bullying, and humiliation form the affective background against which academic and professional achievement become necessary for survival. The Atuda and the university are described not only as institutional tracks but as spaces that “tempered” him and prepared him for life. Beni contrasts the ethnic signification of surnames “Buzaglo or Cohen” with the apparent neutrality of grades (Ariel et al., 2015; Rubinstein & Brenner, 2011). The grade becomes a meritocratic shield: an apparently objective measure that promises to speak louder than ethnic marking and to protect him from being read primarily through surname, origin, or classed background (Ariel et al., 2015; Stevens & Connelly, 2024). To Beni, achievement offers a way to become professionally legible through performance rather than through origin. Yet this shield is ambivalent. It enables recognition through performance, but it also reveals the conditional nature of belonging: legitimacy must be repeatedly secured through measurable excellence. Beni’s account therefore shows how professional metrics can function as both liberation and discipline. They allow him to imagine recognition beyond ethnicity while also requiring him to prove himself through measurable achievement (Brøgger & Andersen, 2024).

Across these narratives, compensatory professionalism emerges as mechanism that operates at the intersection of vulnerability, aspiration, ethnic hierarchy, and the desire for secure legitimacy. Achievement is never only about aspiration. It functions as a communicative shield in which success and defense become intertwined. Yoav and Beni show how excellence and “neutral” metrics may be used to heal social wounds or override ethnic marking, while Shmuel and Daniel frame brilliance, credentials, and educational mobility as intergenerational armor against historical barriers. Ultimately, this mechanism exposes the paradox of conditional belonging: participants achieve high organizational legitimacy but often only by producing intensified evidence that they were worthy of recognition in the first place.

## 4. Discussion

This study reconceptualizes organizational belonging as a communicative accomplishment sustained through what we term subterranean labor, thereby shifting analytical attention from formal inclusion policies to the invisible interactional work required of marked employees. This argument extends scholarship that understands inclusion and belonging as relational, affective, and interactional processes rather than merely as formal organizational outcomes (Roberson, 2006; Shore et al., 2011; Izak et al., 2024). For marked subjects, professional legibility is secured through three interrelated mechanisms: calibration, hyphenation, and compensation. While existing scholarship often conceptualizes identity management through strategic disclosure, authenticity, or impression management, these mechanisms point to the ongoing internal negotiations and energetic costs that occur before identity becomes interactionally visible. These mechanisms transform everyday workplace interactions into sites of agency but also into sites of intense self-regulation. Rather than treating belonging primarily as the employee’s capacity to adapt, this study foregrounds the organization’s responsibility to recognize and interpret differences. Participants often achieved professional legitimacy, yet this legitimacy was conditional: it depended on their ability to read organizational cues, adjust their self-presentation, and make themselves intelligible within dominant norms of professionalism. This study contributes to organizational communication scholarship by shifting attention from whether marked employees are included to the communicative labor required for them to become recognizable as legitimate organizational subjects.

The current study distinguishes between the external and internal dimensions of this labor. Identity calibration refers to the external tuning of visibility, language, tone, and bodily conduct in response to organizational cues. Hyphenated subjectivity, by contrast, refers to the internal dialogue through which participants negotiate the relationship between gayness and Mizrahiness before, during, and after workplace interactions. Building on intersectional, queer, and Mizrahi scholarship, we define the “hyphen” not as a fixed intersection between two identity categories but as a relational process through which different aspects of the self mutually authorize, moderate, or translate one another in order to sustain professional legitimacy within organizational life. In the Israeli context, the hyphen is especially significant because Mizrahi identity has historically been negotiated through processes of ethnic classification, spatial marginalization, and struggles over cultural legitimacy (Shohat, 2002; Hartal & Sasson-Levy, 2018; Abutbul-Selinger, 2022; Shem-Tov, 2025). Crucially, participants do not mobilize gayness or Mizrahiness as whole and stable identities. Rather, they draw on particular traits, signs, cultural codes, bodily styles, speech patterns, forms of humor, emotional registers, or relational expectations associated with each identity, depending on what the interactional setting makes possible, useful, or risky.

In this sense, the hyphen is not simply a sign of double marginalization. It is a dynamic communicative formation through which participants make themselves readable, acceptable, and professionally coherent.

This is particularly evident in the ways participants described gayness and Mizrahiness as mutually modifying categories. In some accounts, Mizrahiness softened the perceived elitism or Ashkenazi coding of gayness; in others, gayness enabled forms of intimacy, humor, or emotional proximity that might otherwise have been read as threatening. Similarly, Mizrahiness sometimes functioned as embodied cultural knowledge, allowing participants to establish trust, recognize social codes, and communicate with workers “at eye level”. These findings show that organizational belonging cannot be understood through sexuality or ethnicity separately. Rather, participants experienced workplace interactions through the simultaneous readability of both identities, revealing how organizational norms classify workers through multiple symbolic registers.

The subterranean labor required to operate these mechanisms carries a significant human and energetic cost. We identify four interrelated forms of work that compose this hidden effort: vigilance regarding organizational cues, interpretation of subtle subtexts, adaptive self-presentation, and protective labor against devaluation. These dynamics resonate with studies of organizational inequality showing that marked workers are often required to manage visibility, affect, and professional credibility in ways that remain institutionally unrecognized (Sasson-Levy & Shoshana, 2013; Tsalach, 2013; Karazi-Presler, 2021; Maji et al., 2024; Stevens & Connelly, 2024). Compensatory professionalism, namely the intensified investment in excellence, often functioned as the ultimate shield against misrecognition. Importantly, compensatory professionalism is not merely an outcome of organizational pressure but constitutes a communicative strategy through which participants seek to pre-empt misrecognition. Yet this also reveals the paradox of conditional belonging: the ability to “play the game” and survive within the organization should not be confused with genuine safety. Adaptation may indicate not that the organization is inclusive but that the worker has become highly skilled at carrying a burden the organization does not see.

Methodologically, this study shows the value of following participants’ narratives rather than beginning from predefined identity categories. Such an approach makes visible forms of organizational labor that are often difficult to capture through survey-based research. Because participants were invited to narrate how they move through workplace interactions, the analysis could trace not only whether identities were disclosed or concealed but which elements of those identities were emphasized, softened, withheld, or translated in specific situations. This narrative approach therefore reveals the micro-communicative work through which marked subjects produce belonging under conditions of unequal recognition.

For DEI practice, these findings suggest a move from surface-level recognition toward subterranean attentiveness. Such attentiveness requires organizations not merely to diversify their workforce but to reconsider the communicative norms through which competence, professionalism, and legitimacy are recognized. This argument contributes to critiques of diversity initiatives that remain focused on representation while leaving dominant organizational norms intact and to recent work showing that DEI outcomes depend on employees’ perception of legitimacy, engagement and organizational commitment (Roberson, 2006; Shore et al., 2011; Brøgger & Andersen, 2024; Showkat & Yahya, 2025). A productive organizational environment is one that notices the nuanced labor employees perform and actively works to reduce the communicative burden placed on marked subjects. By creating conditions in which difference can exist without constant self-protection or over-performance, organizations can move from demanding assimilation toward enabling employees to expend less energy on being legible and more on being full participants in organizational life. Importantly, subterranean labor should not be understood solely as an expression of constraint. It also reflects creativity, strategic competence, and the capacity of marginalized workers to transform socially assigned meanings into resources.

Subterranean labor therefore extends existing approaches to identity work by emphasizing the invisible communicative effort required to anticipate, interpret, and manage how others will read one’s identity within unequal organizational contexts. 

More broadly, this study suggests that organizational inequality is reproduced not only through formal exclusion but through ordinary communicative expectations that remain largely invisible. By locating inequality in the everyday practices through which legitimacy is produced, the study reframes belonging not as a condition organizations simply grant employees but as a relational achievement that organizations must learn to sustain.

### Practical Implications for DEI and Workplace Communication

Our findings suggest that organizations must move beyond prevalent firm-level DEI paradigms that focus primarily on formal representation and instead dedicate attention to the everyday communicative norms that determine professional legitimacy. One practical implication is the need for what might be termed a “code audit”: a systematic examination of the unwritten definitions of professionalism that shape evaluation, promotion, and belonging. This involves questioning whether specific linguistic patterns—such as the “white/academic language” described by Shmuel are essential to role performance or function as invisible cultural barriers that impose subterranean labor on Mizrahi employees.

Furthermore, in an increasingly digitalized workplace, organizations should grant employees autonomy over their digital cues. Allowing workers to manage the visibility of their names or backgrounds on platforms like Zoom should be viewed as a legitimate strategy for maintaining focus on professional performance rather than a lack of commitment. This does not mean that organizations should encourage concealment but that they should recognize how digital infrastructures can expose workers to unwanted ethnic, classed, or biographical questioning. Digital autonomy can therefore reduce unnecessary identity labor and allow professional interaction to remain focused on the work itself. By institutionalizing subterranean attentiveness, organizations can reduce the need for marked subjects to continuously calibrate their identities for the sake of organizational legibility.

Secondly, organizations must actively work to reduce the “Minority Tax” and the cumulative allostatic load placed on gay-Mizrahi employees. Our analysis shows that being an “LGBTQ+ Expert”, a role in which employees like Yahli strategically mediate their identity to foster trust, is a skilled achievement that carries a heavy energetic cost. To mitigate this, managers should foster identity-specific support and move toward a model of “cultural humility” that recognizes diverse forms of cultural capital, such as the relational “eye-level” management practiced by Avner. Instead of expecting minority employees to serve as the default educators of the organization, HRM should distribute the burden of inclusion to “allies” through formal mentoring programs. Ultimately, creating psychological safety means ensuring that excellence is not required as a compensatory shield; managers should provide performance-focused feedback that is decoupled from cultural fit to prevent the exhaustion associated with compensatory professionalism. Such feedback should distinguish between actual role performance and culturally coded expectations regarding speech, affect, masculinity, refinement, or interpersonal style. In this sense, the managerial task is not to help marked workers become better at carrying subterranean labor but to redesign organizational conditions so that less of this labor is required.

Several limitations should be acknowledged. The study focuses on a relatively small group of gay-Mizrahi men in Israel and therefore does not claim statistical generalizability. Since the analysis relies on participants’ retrospective narratives, future research combining ethnographic observation or workplace interaction analysis could further illuminate how subterranean labor unfolds in real time. Moreover, participants who chose to discuss sexuality and ethnicity may represent individuals with heightened reflexivity regarding identity negotiation, meaning that other gay-Mizrahi men may experience similar dynamics without necessarily articulating them. The study’s contribution lies instead in theoretical transferability and conceptual development. Because the study focuses specifically on gay-Mizrahi men in Israel, it does not assume that the mechanisms identified here operate in the same way among other minority employees, nor in other national or cultural contexts. At the same time, the framework may be analytically useful for examining how organizational legibility and subterranean labor are produced in other intersectional locations. Future research may therefore examine whether these processes appear through similar, different, or distinct mechanisms across other groups and settings, and how institutions might reduce, rather than simply benefit from, the hidden labor through which marked subjects make themselves professionally recognizable.

## Data Availability

The data are not publicly available due to privacy and ethical restrictions, as the interview transcripts contain sensitive personal narratives that could compromise participant anonymity. Further inquiries can be directed to the corresponding author.
